# Umbelliferone attenuates cisplatin‐induced acute kidney injury by inhibiting oxidative stress and inflammation via NRF2

**DOI:** 10.14814/phy2.15879

**Published:** 2023-11-29

**Authors:** Zhenle Yang, Ruofei Ning, Qianying Liu, Ruixian Zang, Suwen Liu, Shuzhen Sun

**Affiliations:** ^1^ Department of Pediatrics Shandong Provincial Hospital Affiliated to Shandong First Medical University Jinan Shandong China; ^2^ Nanjing Key Laboratory of Pediatrics Children's Hospital of Nanjing Medical University Nanjing Jiangsu China; ^3^ Department of Pediatrics Shandong University, Shandong Provincial Hospital Jinan Shandong China

**Keywords:** cisplatin‐induced AKI, inflammation, NRF2, oxidative stress, UMB

## Abstract

In this study, we investigated the nephroprotective effects of Umbelliferone (UMB) against cisplatin‐induced acute kidney injury (AKI). C57BL/6J mice were treated with cisplatin via a single intraperitoneal injection (25 mg/kg) with or without UMB (40 mg/kg/day) by gavage. Renal function, apoptosis, oxidative stress, inflammation, and mitochondrial function were analyzed to evaluate kidney injury. In vitro, human proximal tubule epithelial cells were treated with cisplatin, with or without UMB, for 24 h. Western blotting and immunohistochemistry were performed to explore the mechanisms underlying the nephroprotective effects of UMB. Cisplatin‐induced renal dysfunction, including increases in blood urea nitrogen, serum creatinine, and renal tubular injury indices (NGAL and KIM‐1), were significantly attenuated by UMB treatment, along with renal phenotypic changes and renal tubular injury, as evidenced by improved renal histology. Moreover, NRF2 was activated by UMB pretreatment, along with the inhibition of oxidative stress and inflammatory response, as evidenced by decreased levels of antioxidant genes and inflammatory cytokines in cisplatin‐induced AKI. Our results demonstrate that UMB can protect against cisplatin‐induced nephrotoxicity, which is mediated by the NRF2 signaling pathway via antioxidant and anti‐inflammatory activities, suggesting the clinical potential of UMB for the treatment of AKI.

## 1 INTRODUCTION

Acute kidney injury (AKI) is one of the most common complications in acutely ill patients, and its incidence is rapidly increasing worldwide (Mehta et al., n.d.; Hoste et al., 2018). Patients with AKI often exhibit multiple organ dysfunction, associated with considerable morbidity and mortality and high costs (Liu et al., 2020; Ostermann & Cerda, 2018). AKI is mainly caused by sepsis, drug‐induced nephrotoxicity, or ischemia–reperfusion injury (Gameiro et al., 2020; Hoste et al., 2018; Kellum et al., 2021). Nephrotoxicity induced by drugs, such as cisplatin, remains one of the most common causes of AKI in hospitalized patients (Crona et al., 2017; Kim & Moon, 2012). Importantly, AKI is not only associated with short‐term adverse outcomes but can also lead to the development of chronic kidney disease or end‐stage renal disease (He et al., 2017; Kurzhagen et al., 2020; Levey, 2022). In particular, patients with cisplatin‐induced AKI have a significant risk of developing chronic kidney disease after cisplatin treatment as cancer survival rates improve (Sharp et al., 2018). Owing to the complexity of AKI pathogenesis, no specific treatment is available. Therefore, identifying effective renoprotective agents or methods for clinical treatment is necessary to prevent and control AKI.

Oxidative injury and inflammatory perturbations contribute to AKI (Dennis & Witting, 2017; Gong et al., 2020; Ronco et al., 2019). The accumulation of cisplatin in renal cells can lead to the impairment of mitochondrial function and inactivation of glutathione, a well‐recognized cellular antioxidant, subsequently resulting in an imbalance in redox homeostasis and the production of reactive oxygen species (ROS) (Fang et al., 2021; Mapuskar et al., 2021; Perazella, 2019). The overproduction of ROS causes the release of pro‐apoptotic factors, eventually leading to acute tubular necrosis (Mapuskar et al., 2021; Qi et al., 2019). Moreover, cisplatin induces an inflammatory response through the activation of many inflammatory cytokines and chemokines, including nuclear factor kappa B (NF‐κB) and tumor necrosis factor‐alpha (TNF‐α) (Fang et al., 2021; Ozkok et al., 2016; Zhang et al., 2007). The direct and indirect damage induced by cisplatin ultimately exacerbates renal injury and causes functional changes in the kidney (Qi et al., 2019). Nuclear factor erythroid 2‐related factor 2 (NRF2) is an important transcription factor involved in the inhibition of oxidative stress and regulation of the inflammatory response (Ahmed et al., 1863; Guerrero‐Hue et al., 2020; Nguyen et al., 2009). It can regulate antioxidant genes, such as heme oxygenase‐1 (HO‐1) and superoxide dismutase (SOD) (Nguyen et al., 2009; Wang, Zhang, et al., 2020). Furthermore, studies have suggested that NRF2 exerts its anti‐inflammatory effect by inhibiting NF‐κB to control the release of inflammatory cytokines such as TNF‐α and interleukin‐1‐beta (IL‐1β) (Ahmed et al., 1863). Therefore, we speculate that a potent antioxidant and anti‐inflammatory agent acting on NRF2 may be useful in the treatment of AKI.

Umbelliferone (UMB), a benzopyrone belonging to the coumarin family, is commonly found in plants (Seoane‐Rivero et al., 2020). As the main active ingredient of the Chinese herbal medicine, Cortex Fraxini, UMB exhibits a vast array of bioactive properties, such as antibacterial, anti‐inflammatory, antioxidant, antitumor, antiviral, and enzyme‐inhibitory effects (Garg et al., 2020). Owing to its immense pharmacological properties, we hypothesized that UMB may have a protective effect against AKI.

In the present study, we investigated the nephroprotective effect of UMB in a cisplatin‐induced AKI model and clarified the role of the NRF2 signaling pathway and its antioxidant and anti‐inflammatory activities.

## 2 MATERIALS AND METHODS

### 2.1 Animals

Adult male C57BL/6J mice, approximately 8 weeks old, were obtained from the Laboratory Animal Center of Nanjing Medical University (Nanjing, China). The mice were maintained in a standard SPF animal room at constant temperature (25 ± 2°C), humidity (60 ± 10%), and a 12 h light–dark cycle with free access to food and water.

### 2.2 Establishment of mouse model of cisplatin‐induced AKI and UMB treatment

**SCHEME 1:**
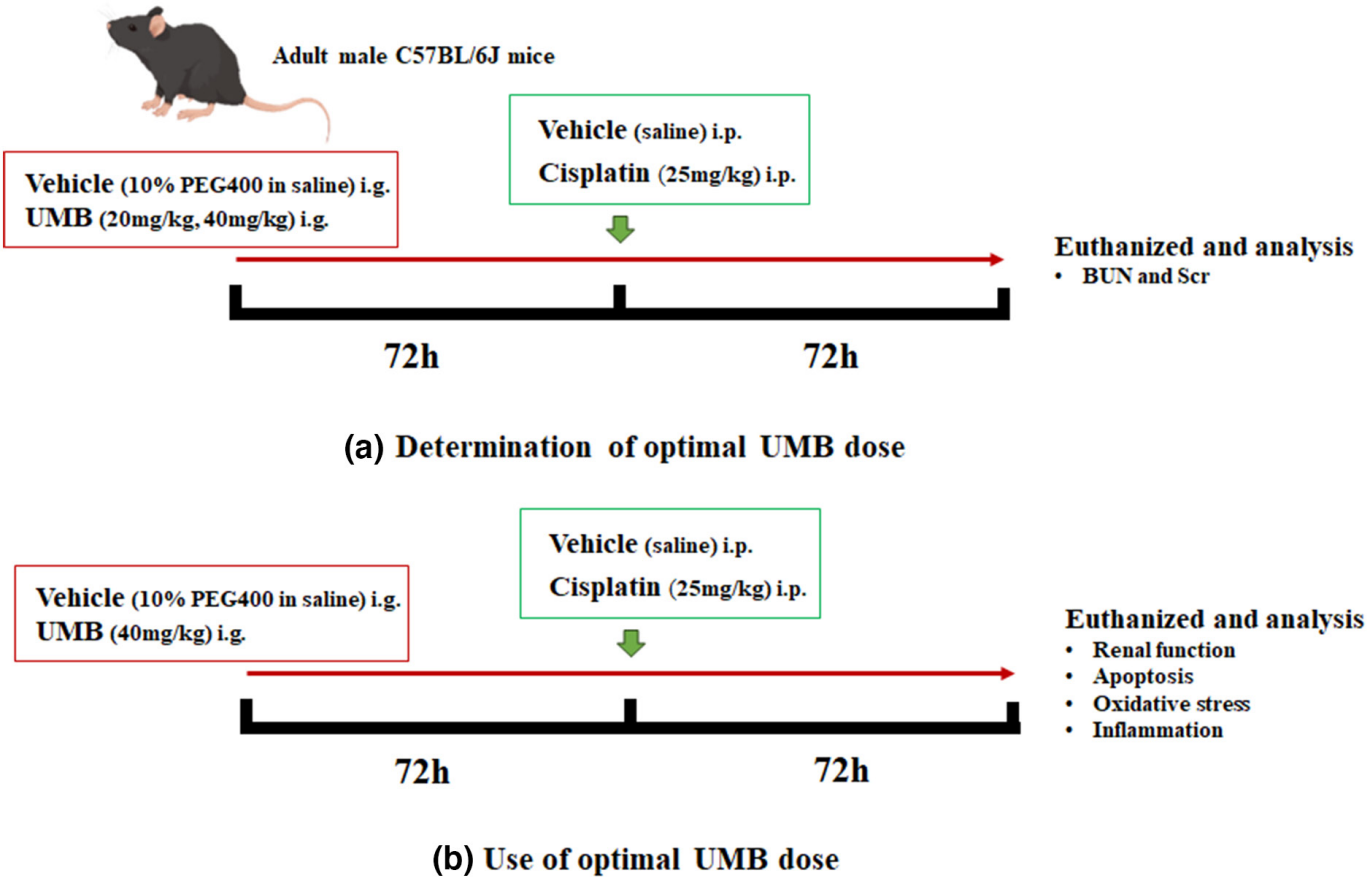
The protocol timeline for UMB treatment in cisplatin‐induced acute kidney injury in mice.

UMB (93–35‐6; Sigma‐Aldrich, St. Louis, MO, USA) at doses of 20 mg/kg and 40 mg/kg was administered to mice for 72 h, followed by cisplatin challenge (a single intraperitoneal (i.p.) injection (25 mg/kg)) for an additional 72 h (UMB treatment was continued once daily). The therapeutic effects of UMB (40 mg/kg) on cisplatin‐induced acute injury were evaluated. Mice were assigned to four groups: vehicle (10% PEG400 in saline; *n* = 6), UMB (*n* = 6), cisplatin (vehicle + cisplatin; *n* = 6), and cisplatin plus UMB (40 mg/kg; *n* = 6). The mice were treated with cisplatin (15663–27‐1; Sigma‐Aldrich) via a single i.p. injection (25 mg/kg) in both the cisplatin and UMB 40 mg/kg + cisplatin groups. The vehicle and UMB groups were administered equal amounts of saline. The UMB treatment groups were treated with 40 mg/kg/d UMB in 10% PEG400 in saline once daily by intragastric (i.g.) administration 72 h before cisplatin treatment and once daily until the mice were sacrificed; the other groups received an equal amount of vehicle (10% PEG400 in saline) once daily. Mice in all groups were euthanized by CO_2_ inhalation (30%) after 72 h of cisplatin treatment (Shomer et al., 2020). The protocol timeline for UMB treatment in mice is shown in Scheme 1. Serum and kidney samples were obtained and stored at −80°C for further analysis. Tissues collected for histological analysis were fixed with 4% paraformaldehyde. The remaining kidney tissues were stored at −80°C for mRNA and protein analyses. All animal experiments were approved by the Institutional Animal Care and Use Committee of Nanjing Medical University (registration number: IACUC 14030112–2).

### 2.3 Cell culture and treatment

Human proximal tubule epithelial cells (HK2) were obtained from American Type Culture Collection (ATCC Manassas, VA, USA). The cells were cultured in Dulbecco's modified Eagle's medium (DMEM)/F‐12 medium (Gibco, 319‐075‐CL) supplemented with 10% fetal bovine serum (FBS; GIBCO, 26170035), penicillin (100 U/mL), and streptomycin (100 μg/mL). The cells were maintained at 37°C in a humidified incubator (5% CO_2_), cultured without FBS after reaching 70% confluence, and pretreated with UMB (40 μg/mL) dissolved in dimethylsulfoxide (DMSO) for 4 h. The HK2 cells were thereafter treated with cisplatin (10 μg/mL) for 24 h.

### 2.4 Renal function

Blood samples were collected from the inferior vena cava vein of mice (approximately 0.2 mL) after anesthesia and centrifuged at 1500 rpm for 20 min at room temperature to obtain serum samples for analysis. Heparin was used as the anticoagulant. To assess renal function, serum creatinine (Scr) and blood urea nitrogen (BUN) levels were evaluated using an automatic biochemical analyzer (Hitachi 7600 Modular Chemistry Analyzer; Hitachi Ltd., Santa Clara, CA, USA) at the Children's Hospital of Nanjing Medical University.

### 2.5 Quantitative real‐time PCR (qRT‐PCR)

Total RNA was extracted from kidney tissues using TRIzol (9108; TAKARA, Dalian, China), following the manufacturer's instructions. cDNA was obtained from 1 μg of total RNA using a Reverse Transcriptase M‐MLV Kit (TAKARA, 2641A). The primers (Table 1) were designed and synthesized by Generay Biotech (Shanghai, China). Real‐time PCR amplification was performed using SYBR Green Master Mix (q111‐02/03; Vazyme, Nanjing, China) and a QuantStudio 3 Real‐time PCR System (Applied Biosystems, Foster City, CA, USA). The cycling conditions were 95°C for 10 min, followed by 40 cycles of 95°C for 15 s and 60°C for 1 min. β‐Actin was used as an internal control and the relative threshold cycle values (ΔCt) were used to analyze the relative levels of messenger RNA (mRNA) expression. Values were converted to fold changes using the 2^−ΔΔCt^ method, as previously described (Rajeevan et al., 2001).

**TABLE 1:** Primer Sequences.

Gene	Primer sequence (5′‐3′)
Mouse NGAL	F: GCAGGTGGTACGTTGTGGG R: CTCTTGTAGCTCATAGATGGTGC
Mouse KIM‐1	F: ACATATCGTGGAATCACAACGAC R: ACTGCTCTTCTGATAGGTGACA
Mouse Bax	F: TGGAGATGAACTGGACAGCAATAT R: GCAAAGTAGAAGAGGGCAACCAC
Mouse BCL2	F: CTCAGGCTGGAAGGAGAAGAT R: AAGCTGTCACAGAGGGGCTAC
Mouse HO‐1	F: AAGCCGAGAATGCTGAGTTCA R: GCCGTGTAGATATGGTACAAGGA
Mouse SOD2	F: CAGACCTGCCTTACGACTATGG R: CTCGGTGGCGTTGAGATTGTT
Mouse mtDNA	F: CACCAATTCCCAACTTATCTGACA R: ACTTGGGACAGGCACATGAC
Mouse 18 s	F: TTCGGAACTGAGGCCATGATT R: TTTCGCTCTGGTCCGTCTTG
Mouse mtATP8	F: ACATTCCCACTGGCACC R: GGGGTAATGAATGAGGC
Mouse PGC‐1α	F: TATGGAGTGACATAGAGTGTGCT R: CCACTTCAATCCACCCAGAAAG
Mouse IL‐1β	F: ACTGTGAAATGCCACCTTTTG R: TGTTGATGTGCTGCTGTGAG
Mouse TNF‐α	F: TCCCCAAAGGGATGAGAAG R: CACTTGGTGGTTTGCTACGA
Mouse β‐Actin	F: GAGACCTTCAACACCCCAGC R: ATGTCACGCACGATTTCCC

### 2.6 Western blotting

Kidney tissues or cells were lysed in protein lysis buffer (50 mM Tris–HCl, 250 mM NaCl, 0.5% Triton X‐100, 50 mM NaF, 2 mM EDTA, and 1 mM Na_3_VO_4_) supplemented with a 1× protease inhibitor cocktail (04693132001; Roche, Basel, Switzerland) for 20 min on ice and centrifuged for 15 min at 4°C and 12,000 rpm. Protein concentration was measured using the Bradford method. Then, 30 μg of total protein was used for western blotting following standard methods. Primary antibodies against cleaved caspase‐3 (1:1000, 9664; Cell Signaling Technology, Danvers, MA, USA), neutrophil gelatinase associated lipocalin (NGAL, 1:1000, ab63929; Abcam, Cambridge, UK), Bax (1:1000, no. 50599‐2‐Ig; Proteintech, Rosemont, IL, USA), NRF2 (1:1000, no. 16396‐1‐AP; Proteintech), SOD2 (1:1000, no. 66474‐1‐Ig; Proteintech), ATPB (1:1000, DF12111; Affinity Biosciences, Cincinnati, OH, USA), GAPDH (1:1000, no. 60004‐1‐Ig, Proteintech), and β‐actin (1:1000, no. 66009‐1‐Ig; Proteintech) were diluted in 3% nonfat milk prepared in TBST (Tris‐buffered saline, 0.1% Tween 20). Peroxidase‐conjugated goat anti‐rabbit secondary antibodies (A0208; Beyotime, Haimen, China) and anti‐mouse secondary antibodies (A0216; Beyotime) were used at a 1:1000 dilution. The enhanced chemiluminescence detection system (Bio‐Rad, Hercules, CA, USA) was used to detect bands. Densitometric analysis was performed using ImageJ software (Wayne Rasband National Institutes of Health, Bethesda, MD, USA).

### 2.7 Kidney histology

Kidney tissues were fixed with 4% paraformaldehyde for 24 h and embedded in paraffin wax. Paraffin sections (3–5 μm) were stained with hematoxylin and eosin (HE). Renal histological analyses were performed by a pathologist in a blinded manner. pathologist. Histological changes were assessed by calculating the percentage of renal tubules showing cell necrosis, tubular dilation, and cast formation. A score from 0 to 4 was assigned to indicate tissue pathological damage as follows: 0, no abnormalities; 1+, <25%; 2+, 25%–50%; 3+, 50%–75%; 4+, >75% (Weidemann et al., 2008; Yang et al., 2020). The sections were examined via microscopy and images were captured using an Olympus BX51 microscope (Olympus, Center Valley, PA, USA).

### 2.8 Immunohistochemistry

Kidney tissues were fixed in 4% paraformaldehyde and embedded in paraffin wax. Paraffin sections (3–5 μm) were used for immunohistochemistry, boiled in 300 mL of 1× improved Citrate Antigen Retrieval Solution (P0083; Beyotime) for 1 min, and cooled for 30 min. After the sections were blocked with 3% H_2_O_2_ for 15 min, they were incubated with 10% normal goat serum for 60 min at room temperature, and then incubated with NGAL (1:100, ab63929; Abcam), NRF2 (1:100, no. 16396‐1‐AP; Proteintech), TNF‐α (1:100, no. 60291‐1‐Ig; Proteintech), and NF‐κB (1:100, no. 10745‐1‐AP; Proteintech) for 24 h at 4°C. After washing thrice with TBST buffer, the sections were incubated at room temperature with horseradish peroxidase‐conjugated secondary antibodies for 60 min. After washing thrice with TBST, a DAB kit (ZLI‐9018; ZsBio, Beijing, China) was used to detect the localization of the peroxidase conjugates.

### 2.9 TUNEL assay

In situ cell death was measured using a Servicebio Fluorescein (FITC) TUNEL Cell Apoptosis Detection Kit, according to the manufacturer's instructions (G1501‐50 T; Servicebio, Wuhan, China). Apoptotic cells were counted, and images were acquired using a fluorescence microscope.

### 2.10 Cell counting Kit‐8 assay

Cell viability was analyzed using a Cell Counting Kit (CCK)‐8 assay kit (HY K0301, MCE). Briefly, HK2 cells were cultured in a 96‐well plate to 70% confluence, and treated with UMB (20, 40, or 80 μM) in serum‐free medium for 24 h. Subsequently, 10 μL of the CCK‐8 reagent was added to the medium and cultured for 2 h. Absorbance was measured at 450 nm using a Multiskan FC microplate reader (Thermo Fisher, Shanghai, China).

### 2.11 Cell apoptosis assay

The treated HK2 cells were washed with cold PBS thrice, trypsinized with EDTA‐free trypsin, and double‐stained with annexin V‐FITC and PI using an apoptosis detection kit (BD Biosciences, 556,547, San Diego, CA, USA) according to the manufacturer's instructions. Apoptosis was detected using a flow cytometer (BD Biosciences, FACSCalibur) and the results were analyzed using FlowJo software (TreeStar, Ashland, OR, USA).

### 2.12 Statistical analysis

The data are presented as the mean ± standard deviation (S.D.) and were analyzed using GraphPad Prism 6. Comparisons among all groups were performed using one‐way or two‐way analysis of variance (ANOVA) followed by Tukey's multiple comparison test. Statistical significance was set at *p* < 0.05.

## 3 RESULTS

### 3.1 UMB ameliorated cisplatin‐induced AKI in mice

**FIGURE 1:**
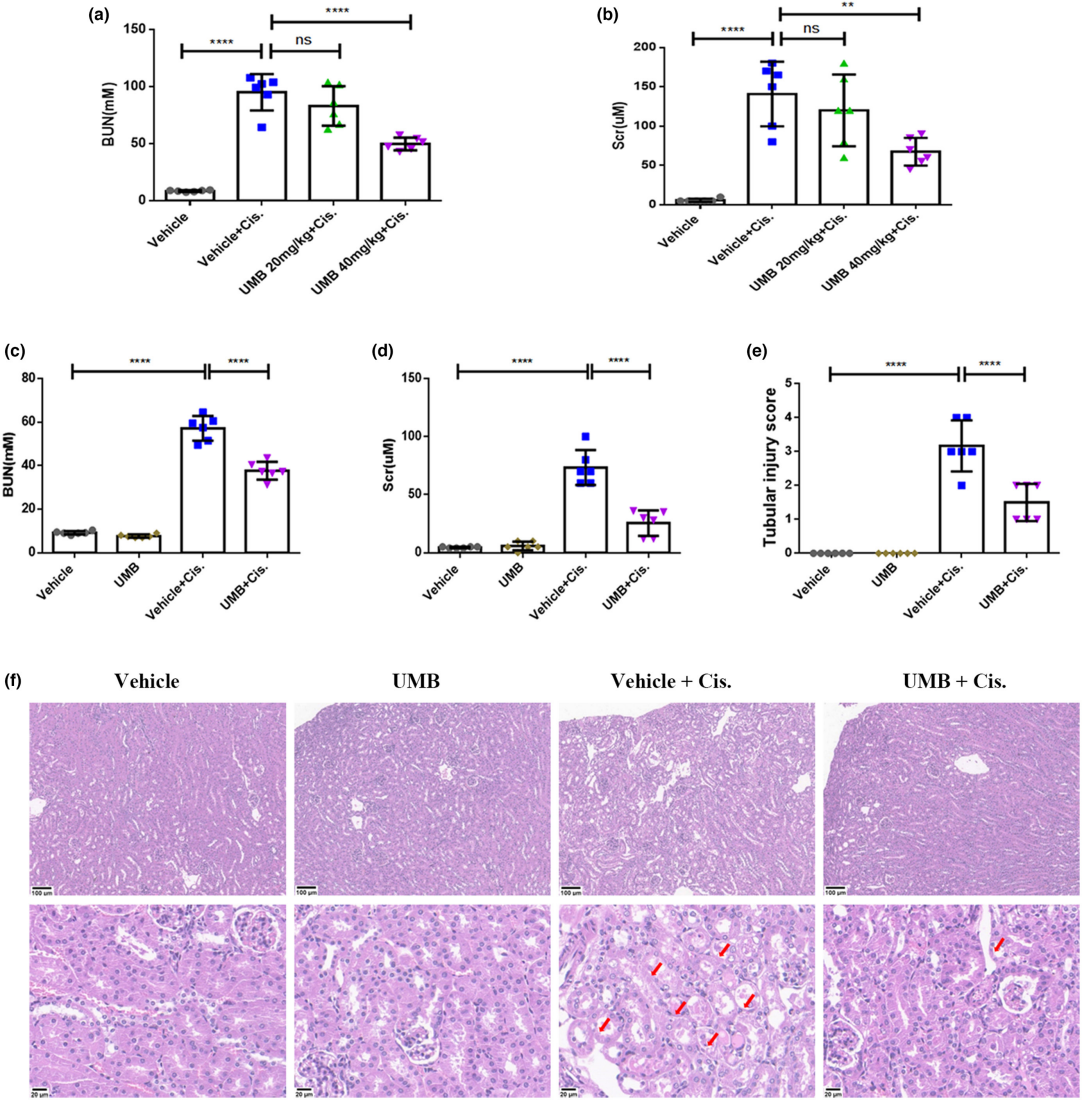
Umbelliferone (UMB) protected against cisplatin‐induced acute kidney injury. (a, b) Blood urea nitrogen (BUN) and serum creatinine (Scr) levels in mice treated with different doses of UMB after 72 h of cisplatin administration (*n* = 6 mice per group). (c, d) Blood urea nitrogen (BUN) and serum creatinine (Scr) levels in mice treated with 40 mg/kg UMB after 72 h of cisplatin administration (*n* = 6 mice per group). (e, f) Representative images of HE staining (magnification: 400×, scale bar: 20 μm) of kidney tissues (40 mg/kg UMB). Red arrow: damaged renal tubules. Data are shown as means ± standard deviation. ****p < 0.0001, **p < 0.01.

**FIGURE 2:**
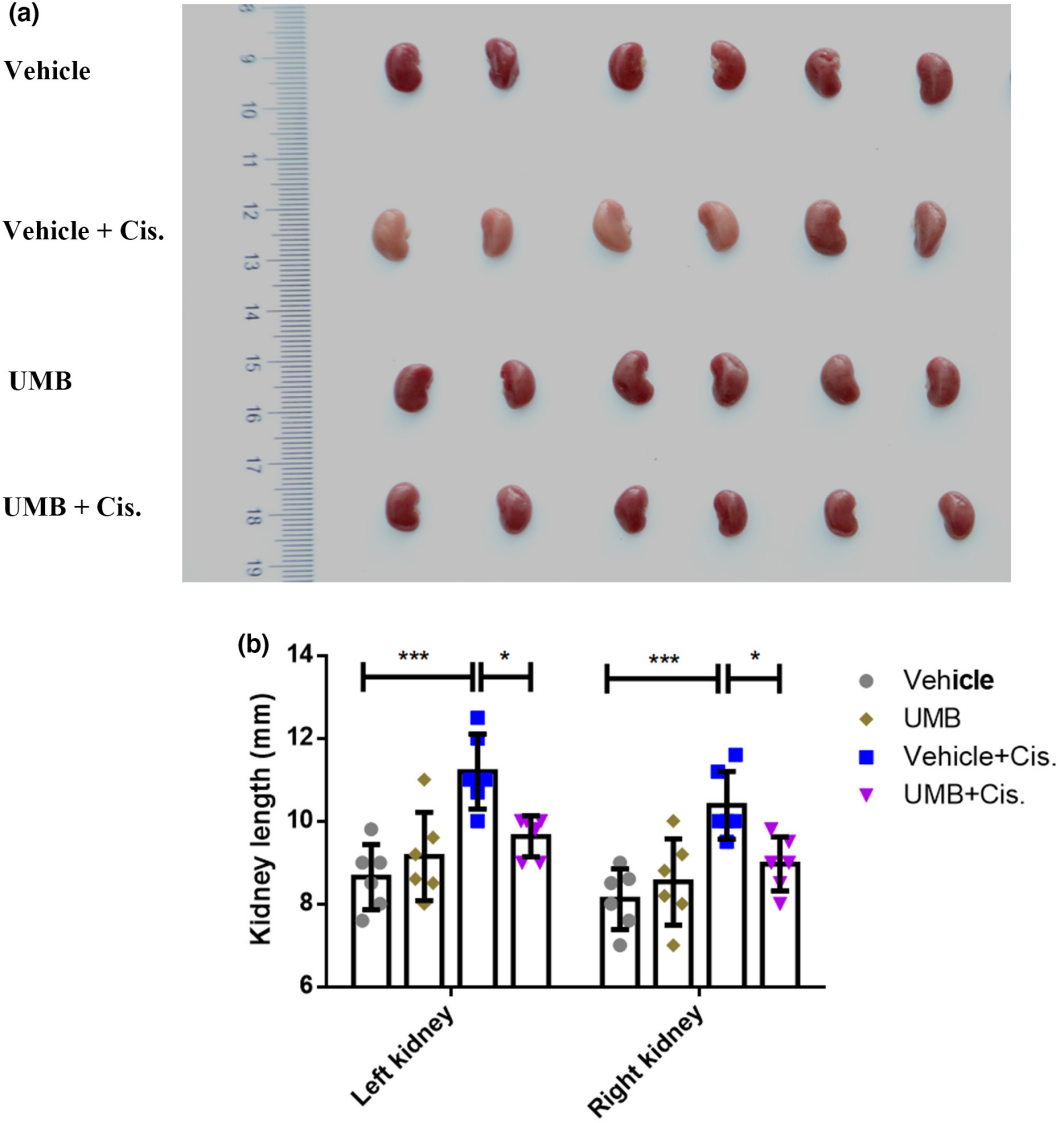
The morphological changes of kidney in mice (UMB 40 mg/kg). (a) Representative images of renal phenotypic changes. (b) The kidney length in mouse from different groups. Data are shown as means ± standard deviation. ***p < 0.001, *p < 0.05.

The effects of UMB on cisplatin‐induced renal dysfunction were analyzed based on BUN and Scr (Tajima et al., 2019). As illustrated in Figure 1a,b, the protective effect of UMB against cisplatin‐induced renal dysfunction was greater at 40 mg/kg than at 20 mg/kg. Thus, mice were pretreated with UMB (40 mg/kg) or vehicle (10% PEG400 in saline) by gavage once daily for three consecutive days before cisplatin administration (Scheme 1). Renal function after 72 h of cisplatin treatment was significantly impaired, as shown by increased BUN (from 9.18 ± 0.32 to 57.17 ± 2.32 mM, *p* < 0.0001; Figure 1c) and Scr (from 4.50 ± 0.22 to 73.33 ± 6.15 μM, *p* < 0.0001; Figure 1d). Renal function was remarkably better after treatment with 40 mg/kg UMB than in the cisplatin group, as evidenced by reduced levels of BUN (from 57.17 ± 2.32 to 37.67 ± 1.68 mM, *p* < 0.0001; Figure 1c) and Scr (from 73.33 ± 6.15 to 25.50 ± 4.44 μM, *p* < 0.0001; Figure 1d). HE staining showed significant tubular injury, mainly manifested as necrosis of tubular epithelial cells, dilation of renal tubules, loss of brush‐borders, and protein cast formation (Figure 1e,f). These cisplatin‐induced renal morphological abnormalities were significantly alleviated by UMB treatment (Figure 1e,f). Finally, compared with the appearance of the kidney in the vehicle group, the kidneys in the cisplatin‐treated group exhibited enlargement and were paler with an uneven surface. Following UMB treatment, the kidneys recovered to a reddish‐brown color and displayed a reduced size compared with their previous state (Figure 2a,b). However, the UMB group did not exhibit significant morphological and pathological changes compared with the vehicle group (Figure 1e,f; Figure 2a,b).

**FIGURE 3:**
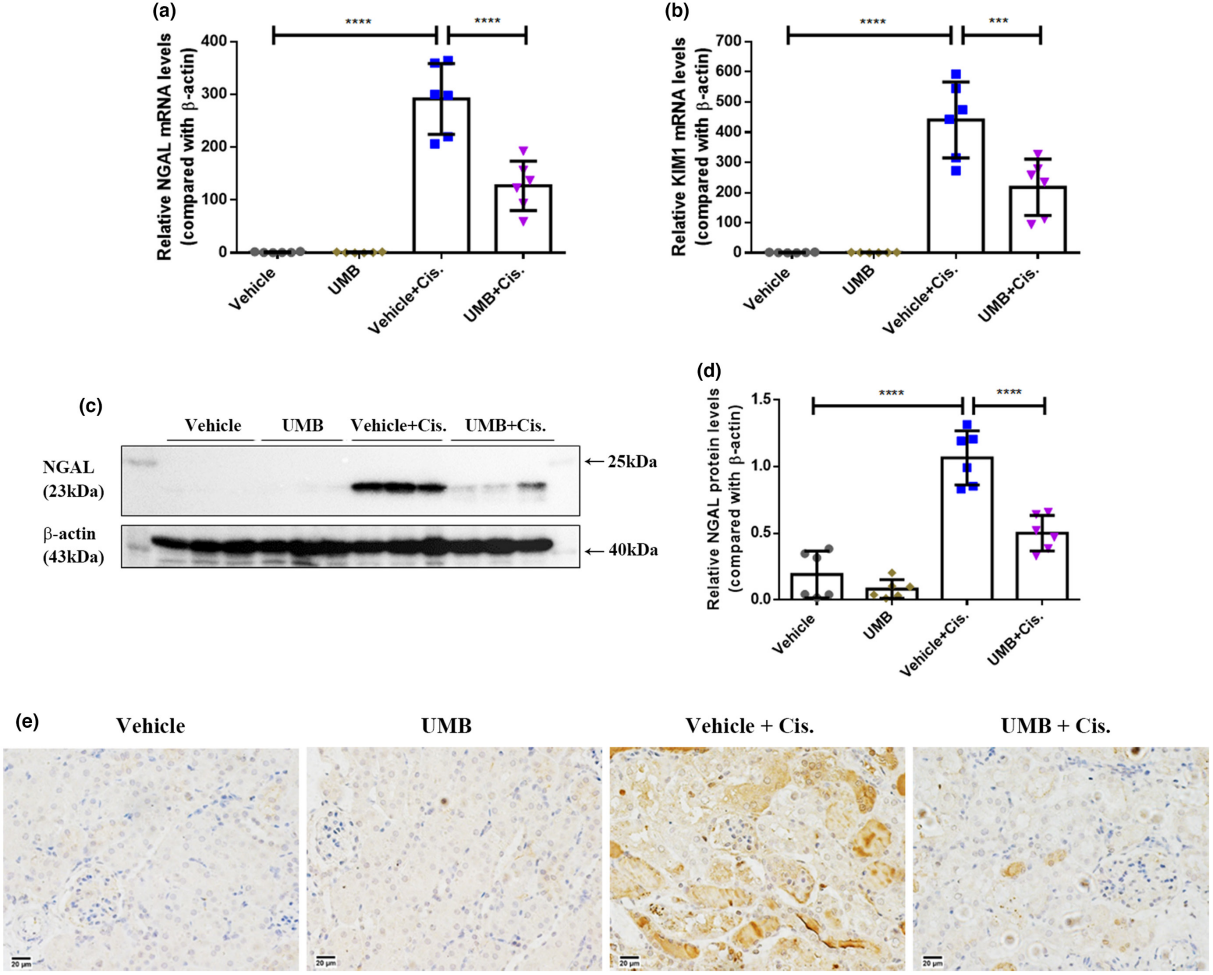
The upregulation of neutrophil gelatinase‐associated lipocalin (NGAL) and kidney injury molecule‐1 (KIM‐1) expression induced by cisplatin was decreased by UMB (40 mg/kg). (a, b) Quantitative PCR was performed to analyze mRNA levels of renal NGAL and KIM‐1. (c) Western blot analysis of NGAL levels in the kidneys of each group of mice. (d) NGAL densitometry analysis. (e) Representative images of immunohistochemistry staining of NGAL in kidneys from different groups (magnification: 400×, scale bar: 20 μm) after 72 h of cisplatin administration. Data are shown as means ± standard deviation (*n* = 6 mice/group). All experiments were duplicated three times. ****p < 0.0001, ***p < 0.001.

To further clarify the renoprotective effects of UMB against cisplatin‐induced AKI, the mRNA levels of the renal tubular injury markers neutrophil gelatinase‐associated lipocalin (NGAL) and kidney injury molecule 1 (KIM‐1) were examined. Quantitative real‐time PCR showed that NGAL and KIM‐1 levels were significantly higher in the kidneys of mice treated with cisplatin than those in the control group, and these increases were attenuated by treatment with 40 mg/kg UMB (Figure 3a,b). Additionally, western blotting and immunohistochemical staining showed that the protein levels of NGAL were significantly higher in the damaged tubules of mice treated with cisplatin than in the control group, and that the cisplatin‐induced changes were significantly attenuated by UMB treatment (Figure 3c–e). It is worth mentioning that no significant disparity was observed between the vehicle and UMB groups. These data suggest that UMB attenuates the loss of renal function and renal tubular injury in cisplatin‐induced AKI mice. Furthermore, based on a comprehensive analysis of morphology, pathology, and renal tubular injury, we did not find any obvious side effects of UMB on the kidneys of normal mice, such as renal enlargement and pallor, tubular dilatation, and tubular epithelial cell necrosis.

### 3.2 UMB attenuated cisplatin‐induced apoptosis in mouse kidneys

**FIGURE 4:**
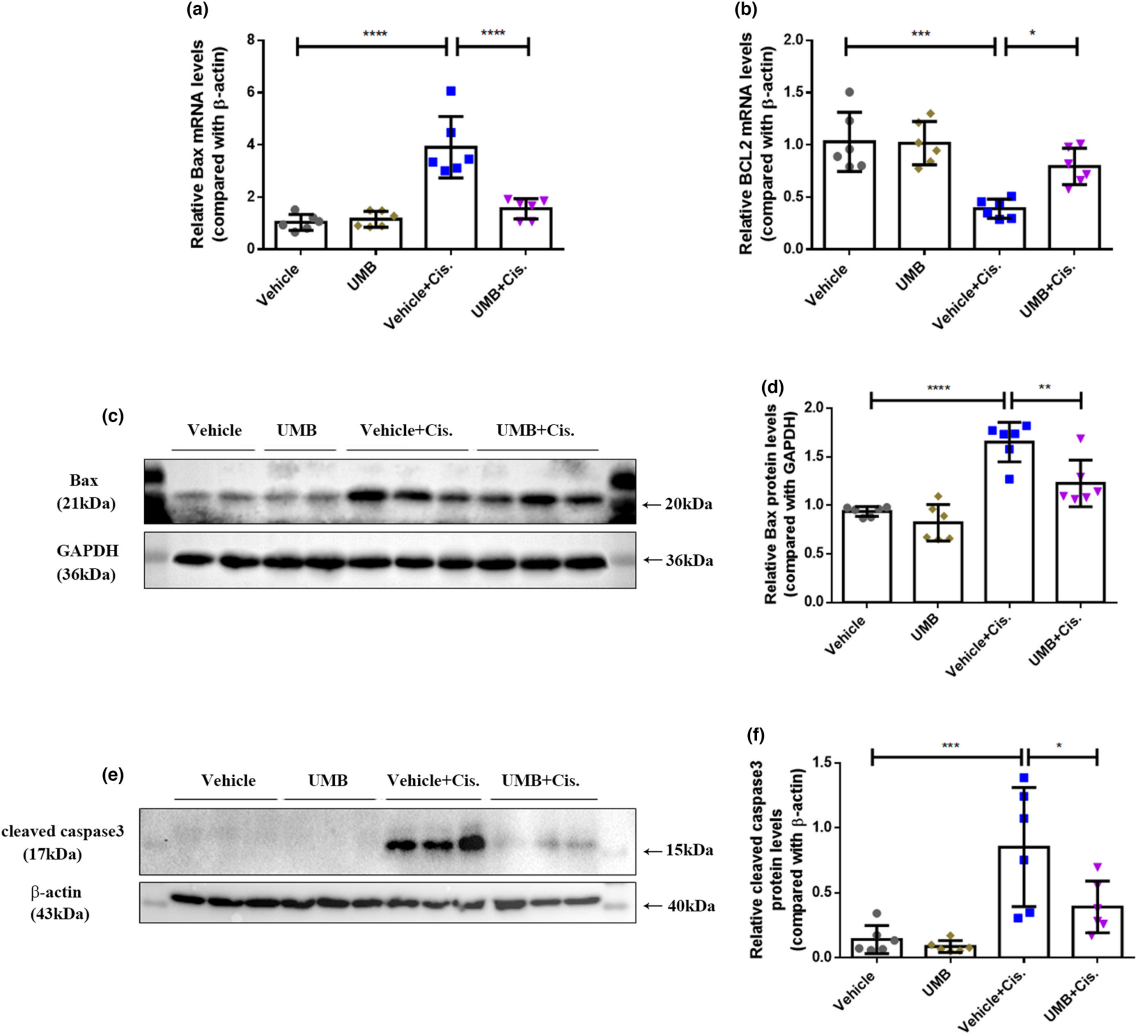
UMB (40 mg/kg) attenuated apoptosis in the kidneys of mice treated with cisplatin. (a) Quantitative PCR was performed to analyze mRNA levels of renal Bax. (b) Quantitative PCR was performed to analyze mRNA levels of renal BCL2. (c) Western blot analysis of Bax levels in the kidneys of each group of mice. (d) Bax densitometry analysis. (e) Western blot analysis of Cleaved caspase3 levels in the kidneys of each group of mice. (f) Cleaved caspase3 densitometry analysis. Data are shown as means ± standard deviation (*n* = 6 mice/group). All experiments were duplicated three times. ****p < 0.0001, ***p < 0.001, **p < 0.01, *p < 0.05.

**FIGURE 5:**
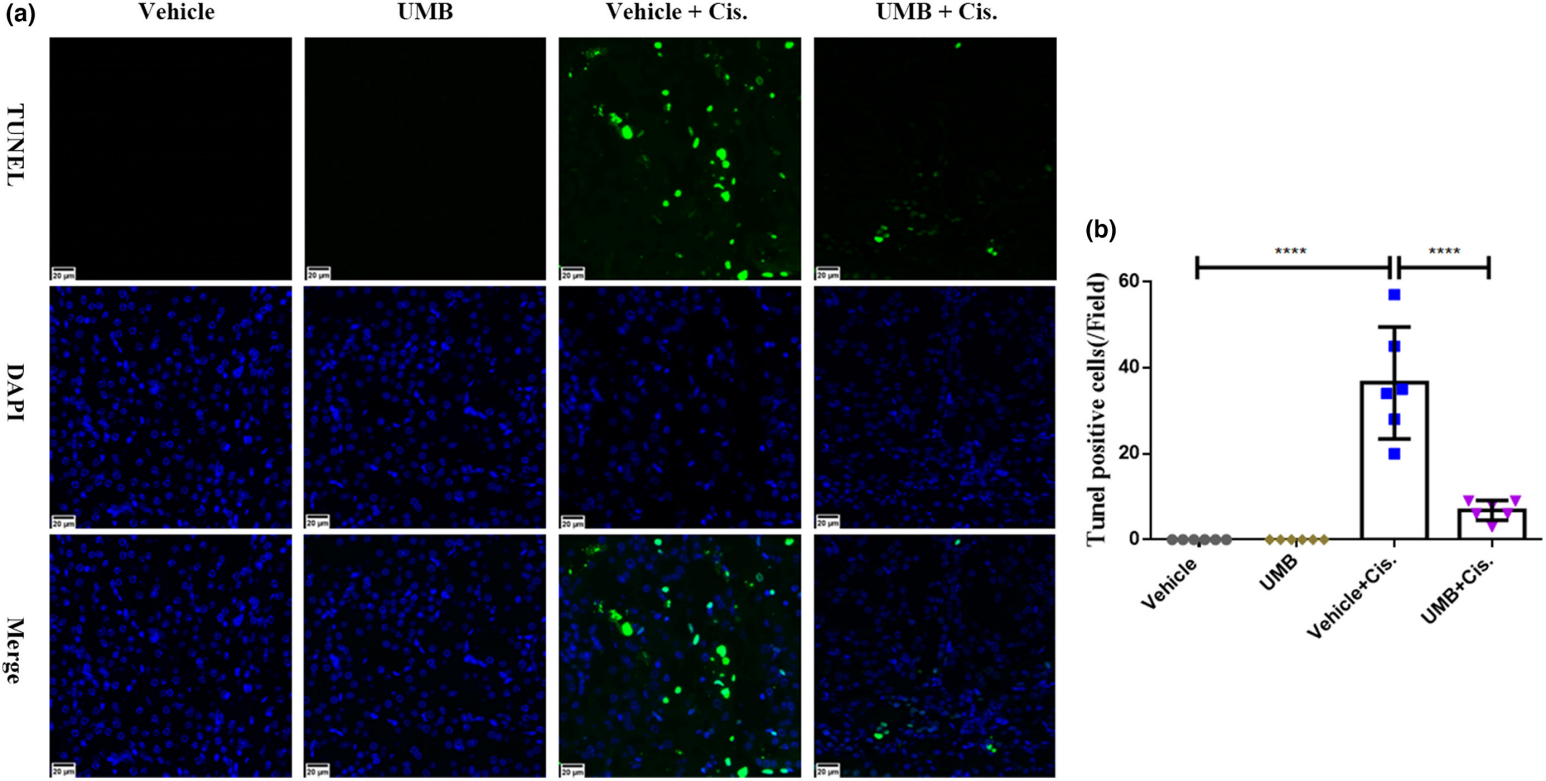
UMB (40 mg/kg) attenuated apoptosis in the kidneys of mice treated with cisplatin. (a) Representative images of TUNEL staining of kidney slides (magnification: 400×, green: TUNEL and blue: DAPI). (b) TUNEL‐positive cells in five random fields from each kidney. Data are shown as means ± standard deviation (*n* = 6 mice/group). ****p < 0.0001.

We analyzed apoptosis in the kidneys of cisplatin‐induced AKI mice. As shown in Figure 4a,c,d, the mRNA and protein levels of Bax in the kidneys of cisplatin‐treated mice were markedly higher than those in the vehicle group, while Bax levels were significantly lower in mice treated with the combination of UMB plus cisplatin than in the cisplatin‐treated group. Consistent with the trend in Bax expression, the protein levels of cleaved caspase‐3 in the kidneys of cisplatin‐treated mice were markedly decreased by UMB treatment (Figure 4e,f). The mRNA levels of BCL‐2 in the kidneys decreased in response to cisplatin and were significantly increased by UMB treatment (Figure 4b). TUNEL staining was used to analyze cisplatin‐induced apoptosis. As shown in Figure 5a,b, there were significantly more TUNEL‐positive cells in the renal tubules of cisplatin‐treated mice than in the control group, and TUNEL‐positive cells were markedly decreased by UMB treatment. These findings suggest that UMB treatment can alleviate apoptosis in cisplatin‐induced AKI in mouse kidneys.

### 3.3 Effect of UMB on the expression of NRF2 in mouse kidneys

**FIGURE 6:**
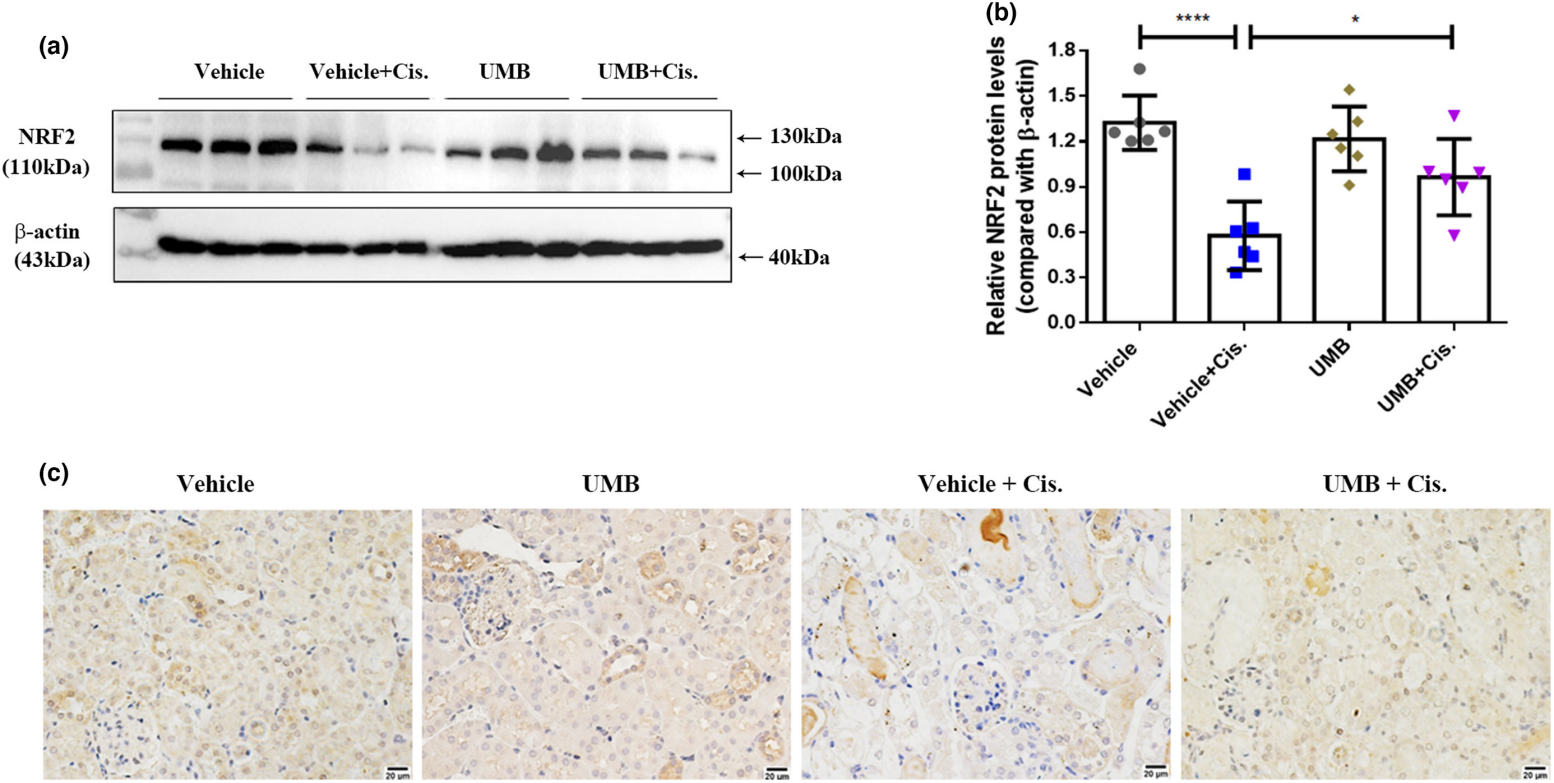
Regulation of UMB (40 mg/kg) on the expression of Nuclear factor erythroid 2‐related factor 2 (NRF2) in the kidneys of mice. (a) Western blot analysis of NRF2 levels in the kidneys of each group of mice. (b) NRF2 densitometry analysis. (c) Representative images of immunohistochemistry staining of NRF2 in kidneys from different groups (magnification: 400×, scale bar: 20 μm). Data are shown as means ± standard deviation (*n* = 6 mice/group). ****p < 0.0001, *p < 0.05.

NRF2 is essential for oxidative stress and the inflammatory response, which play important roles in the progression of cisplatin‐induced AKI (Ahmed et al., 1863; Wei et al., 2020). We speculated that the nephroprotective effects of UMB in the cisplatin‐induced AKI model were achieved via the NRF2 signaling pathway, which exhibits antioxidant and anti‐inflammatory activities. Western blotting and immunohistochemical staining showed that the protein levels of NRF2 in the kidneys decreased significantly after cisplatin treatment and were upregulated by UMB treatment (Figure 6a–c). The protein levels of NRF2 did not differ significantly between the vehicle and UMB groups (Figure 6a–c).

### 3.4 UMB inhibits oxidative stress and inflammatory responses in cisplatin‐induced AKI via NRF2

**FIGURE 7:**
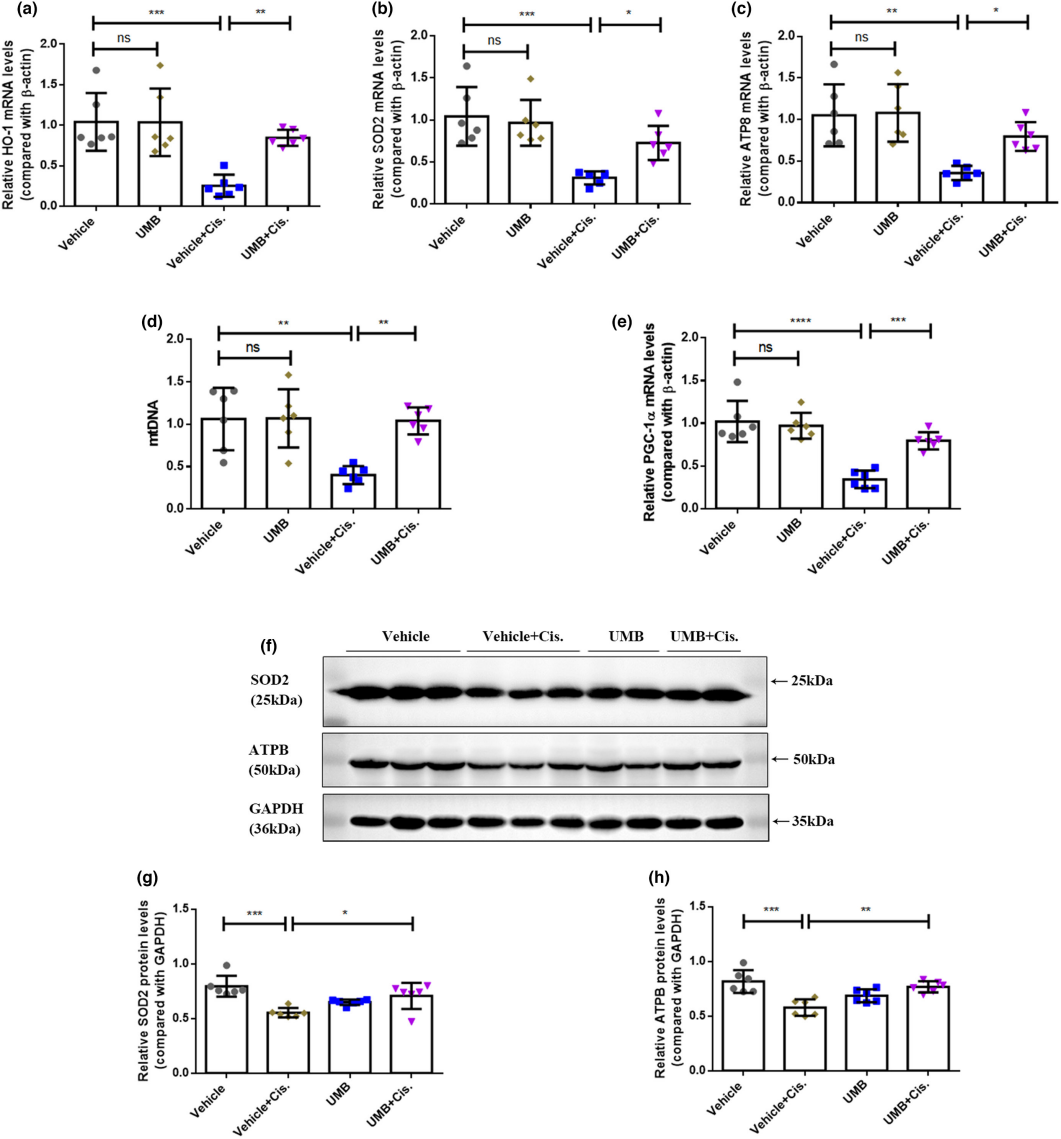
UMB (40 mg/kg) inhibited cisplatin‐induced oxidative stress by acting on NRF2 in mice. (a, b, c) Quantitative PCR was performed to analyze mRNA levels of renal HO‐1, SOD2, and ATP8. (d) Quantitative PCR analysis of mtDNA copy number in the kidneys of mice. 18S rDNA was used as an internal control. (e) Quantitative PCR was performed to analyze mRNA levels of renal PGC‐1α. (f) Western blot analysis of SOD2 and ATPB levels in the kidneys of each group of mice. (g & h) SOD2 and ATPB densitometry analysis. Data are shown as means ± standard deviation (*n* = 6 mice/group). ****p < 0.0001, ***p < 0.001, **p < 0.01, *p < 0.05.

NRF2 can regulate antioxidant genes (Nguyen et al., 2009; Wang, Zhang, et al., 2020). As determined by western blotting and qRT‐PCR, the levels of the antioxidant genes HO‐1, SOD2, and ATPB in the kidneys of mice treated with cisplatin were remarkably lower than those in the control group and significantly increased after UMB treatment (Figure 7a–c,f–h). Furthermore, redox imbalances are closely linked to mitochondrial dysfunction. Accordingly, qRT‐PCR was performed to analyze the mRNA levels of mitochondria‐related genes, including mtDNA copy number and PGC‐1α. The cisplatin‐induced AKI group showed significantly lower expression levels of mtDNA and PGC‐1α than the vehicle group, and the cisplatin‐induced decrease in the expression levels of these genes was partially or completely restored by UMB treatment (Figure 7d,e). Thus, UMB inhibited oxidative stress and improved mitochondrial function in cisplatin‐induced AKI by acting on NRF2.

**FIGURE 8:**
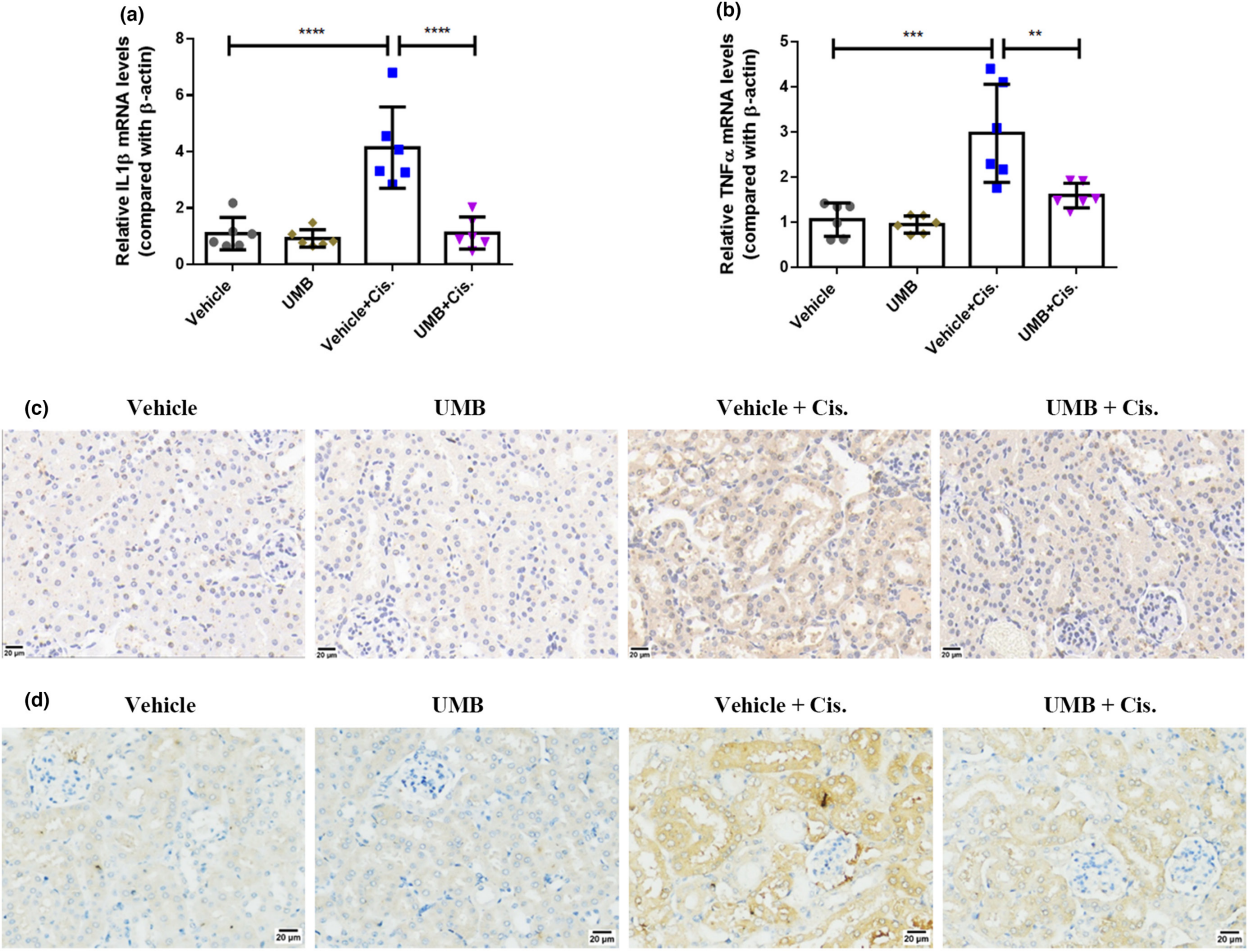
UMB (40 mg/kg) inhibited cisplatin‐induced inflammatory action by acting on NRF2 in mice. (a, b) Quantitative PCR was performed to analyze mRNA levels of renal IL‐1β and TNFα. (c) Representative images of immunohistochemical staining of TNFα in kidneys from different groups (magnification: 400×, scale bar: 20 μm). (d) Representative images of immunohistochemical staining of NF‐κΒ in kidneys from different groups (magnification: 400×, scale bar: 20 μm). Data are shown as means ± standard deviation (*n* = 6 mice/group). ****p < 0.0001, ***p < 0.001, **p < 0.01.

Studies have also suggested that NRF2 exerts anti‐inflammatory effects by inhibiting NF‐κB to control the release of inflammatory cytokines such as IL‐1β and TNF‐α (Ahmed et al., 1863). We analyzed the effects of UMB on cisplatin‐induced renal inflammation. Immunohistochemical staining and qRT‐PCR (Figure 8a–c) revealed that the expression levels of inflammatory factors, including IL‐1β and TNF‐α increased substantially in response to cisplatin, and that these increases were significantly attenuated by UMB. Furthermore, we detected changes in the upstream transcription factor NF‐κB, and immunohistochemical staining showed that UMB effectively mitigated cisplatin‐induced upregulation of NF‐κB (Figure 8d). These findings suggest that UMB treatment attenuates cisplatin‐induced inflammatory activity by upregulating NRF2 expression in the mouse kidney.

### 3.5 UMB protected HK2 cells against cisplatin‐induced apoptosis in vitro via NRF2

**FIGURE 9:**
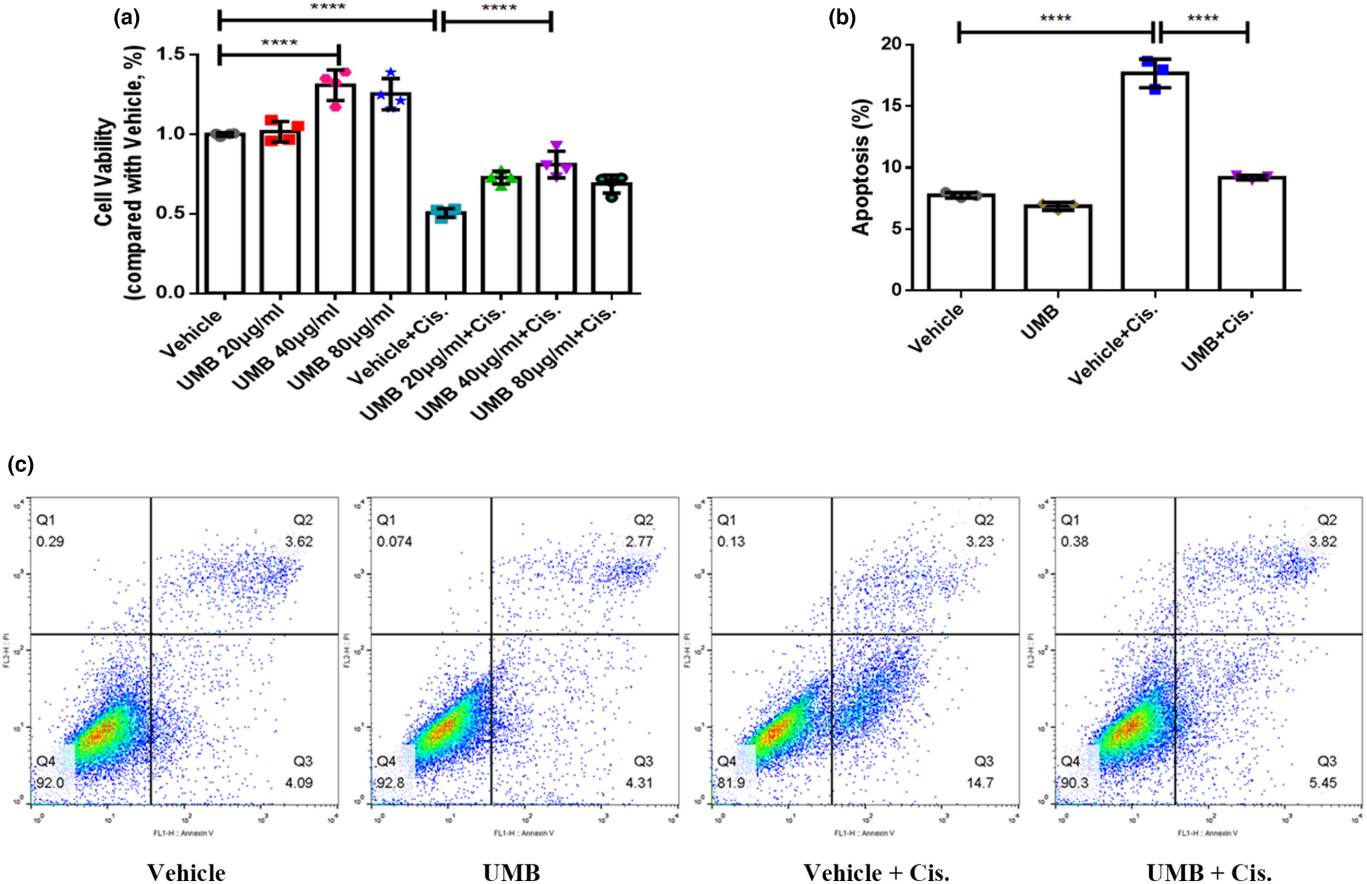
UMB protected Human proximal tubule epithelial cells (HK2) against apoptosis induced by cisplatin in vitro. (a) Cell Counting Kit‐8 assay was performed to analyze cell viability of HK2 cells treated by cisplatin with or without UMB for 24 h at increasing concentrations ranging from 20 to 80 μg/mL. (b) Quantification of flow cytometry. (c) Representative flow cytometry analysis of Annexin V and PI staining. HK2 cells were pretreated with UMB (40 μg/mL) for 4 h, then cisplatin (10 μg/mL) was added and treated for 24 h. All cell experiments were duplicated three times. Data are shown as means ± standard deviation. ****p < 0.0001.

**FIGURE 10:**
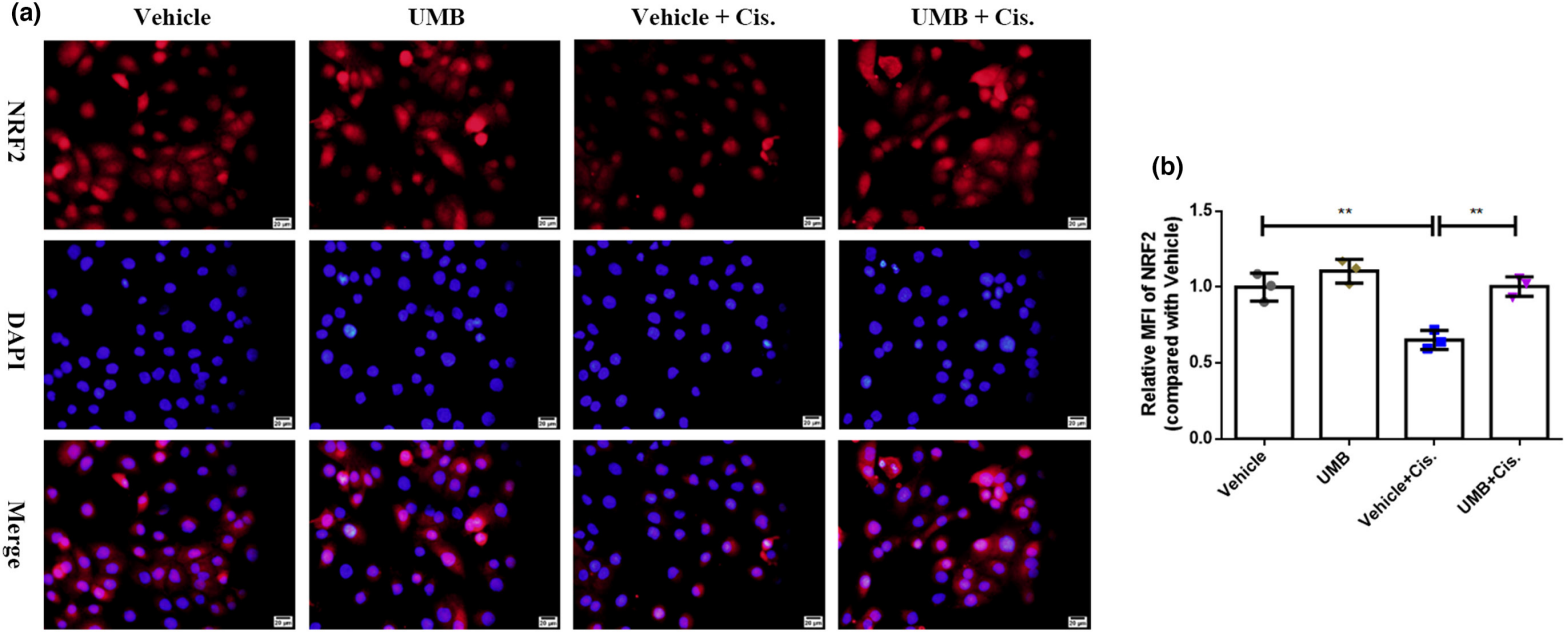
UMB protected HK2 cells by acting on NRF2 in vitro. (a) Representative immunofluorescence analysis of NRF2 with or without UMB (40 μg/mL) administration in the in vitro HK2 cells model (magnification: 400×, scale bar: 20 μm). (b) Graphs showing the results of quantitative analysis of MFI of NRF2 analyzed by ImageJ software. All cell experiments were duplicated three times. Data are shown as means ± standard deviation. **p < 0.01.

To further investigate the protective effect and mechanism of UMB treatment in human proximal tubule epithelial cells in vitro, we measured the changes in apoptosis and the expression and localization of NRF2 in a cisplatin‐induced cell model. The cell viability assay (Figure 9a) suggested that UMB at a concentration of 40 μg/mL had a better protective effect and no obvious toxicity in HK2 cells. Flow cytometry was performed to evaluate the apoptotic response of cisplatin‐induced HK2 cells with or without UMB pretreatment. Throughout the experiment, it was observed that the Vehicle + Cis. group displayed three discernible cell populations: early apoptotic cell‐Q3, late apoptotic cell‐Q2, and normal cell‐Q4. To ensure the precise analysis of these populations, deliberate attempts were made to segregate them into distinct quadrants. Furthermore, the negative control group (vehicle group) predominantly exhibited cell populations in the Q4 quadrant, which ultimately affected the final determination of the gate position. The results showed that the apoptosis of HK2 cells induced by cisplatin was significantly inhibited by pretreatment with 40 μg/mL UMB (Figure 9b,c). In agreement with the protective effect against cell apoptosis, UMB restored the reduced levels of NRF2 expression in response to cisplatin and increased NRF2 nuclear translocation (Figure 10a,b). These results demonstrate that UMB plays a necessary role in protecting against cisplatin‐induced apoptosis in HK2 cells, which may be achieved by the upregulation of NRF2 expression and an increase in NRF2 nuclear translocation.

## 4 DISCUSSION

AKI is characterized by an acute decrease in renal function and is an important risk factor for the development and progression of chronic kidney disease (Gameiro et al., 2020; Kurzhagen et al., 2020). As a main cause of AKI, nephrotoxicity limits the therapeutic application of drugs, especially cisplatin, a first‐line chemotherapy agent (Perazella & Rosner, n.d.; McSweeney et al., 2021; Volarevic et al., 2019). The proposed mechanisms underlying cisplatin‐induced AKI include an imbalance in redox homeostasis, acute tubular necrosis, and inflammatory perturbation, which may ultimately lead to renal injury and functional changes in the kidney (Holditch et al., 2019; McSweeney et al., 2021). The pathogenesis of cisplatin‐induced AKI is complex, early diagnosis is difficult, and clinically effective and specific treatments for preventing or treating cisplatin‐induced nephrotoxicity are lacking.

Many traditional and complementary drugs from natural products with potential antioxidant and anti‐inflammatory properties, including herbs, vitamins, minerals, trace elements, and nutritional supplements, have been developed to protect against cisplatin‐induced AKI (Cohen & Hunter, 2017; Huang et al., 2019; Mercantepe et al., 2018; Rjeibi et al., 2018; Zang et al., 2019). Notably, natural products with potential therapeutic effects on cisplatin‐induced AKI also attenuate kidney diseases caused by other factors (Fang et al., 2021). Therefore, the identification of natural products with high efficacy and low toxicity for the treatment of cisplatin‐induced AKI is important and has broad clinical implications.

As a coumarin compound present in edible plants and fruits, UMB is non‐toxic, safe, and possesses extensive pharmacological properties (Cruz et al., 2020; Liang et al., 2017; Seoane‐Rivero et al., 2020). Recent studies have shown that UMB exerts antioxidant and anti‐inflammatory effects in multiple disease models, including diabetic nephropathy (Wang et al., 2019), colistin or gentamicin‐induced kidney injury (Hassanein, Ali, et al., 2021; Wang, Ishfaq, et al., 2020), ischemia/reperfusion‐induced hepatic injury (Hassanein, Khader, et al., 2021), CCl4‐induced liver fibrosis (Mahmoud et al., 2019), and gastrointestinal diseases (Cruz et al., 2020). In the present study, we demonstrated that UMB ameliorates cisplatin‐induced nephrotoxicity in mice. Our results showed that the impaired renal function induced by cisplatin (e.g., increased BUN and Scr) was attenuated by UMB at doses of 20 mg/kg and 40 mg/kg by oral gavage. However, the effect of 20 mg/kg UMB was not significant. Therefore, we chose 40 mg/kg as the optimal drug dose for further analyses. UMB also attenuated the cisplatin‐induced increases in the renal tubular injury markers NGAL and KIM‐1. Additionally, we observed that cisplatin‐induced changes in renal phenotype, renal tubular morphological damage, apoptosis, inflammation, oxidative stress, and mitochondrial function in mice were attenuated by UMB. Consistent with these findings, results from the in vitro HK2 model also showed a protective effect of UMB treatment against cisplatin‐induced renal tubular cell apoptosis. The findings of the present study further demonstrate that UMB could ameliorate cisplatin‐induced renal tubular cell injury and support the clinical application of UMB in the treatment of cisplatin‐induced AKI.

Although previous studies have demonstrated the protective effects of UMB in different models of kidney disease, including glycerol‐induced myoglobinuric AKI (Kaur et al., 2021), diabetic nephropathy (Wang et al., 2019), and colistin or gentamicin‐induced kidney injury (Hassanein, Ali, et al., 2021; Wang, Ishfaq, et al., 2020), the roles and mechanisms of action of UMB in cisplatin‐induced AKI remain unclear. Preclinical and clinical studies have demonstrated that an increase in NRF2 activity could ameliorate AKI and prevent chronic kidney disease progression (Nezu et al., 2017; Wei et al., 2020). Therefore, we investigated the expression of NRF2, a crucial transcription factor involved in the inhibition of oxidative stress and regulation of the inflammatory response (He, Antonucci, & Karin, 2020; He, Ru, & Wen, 2020). Our results indicate that cisplatin decreased NRF2 levels, whereas UMB increased NRF2 nuclear translocation and restored NRF2 levels to those in the control group. Previous studies have suggested that NRF2 is activated in response to an imbalance in the redox system, is transferred to the nucleus, and forms a heterodimer, ultimately affecting the transcription of antioxidant genes (He, Ru, & Wen, 2020; Wei et al., 2020) and proinflammatory genes (Ahmed et al., 1863; He, Antonucci, & Karin, 2020). Consistent with previous studies, UMB treatment restored NRF2 activity while significantly increasing the levels of antioxidant markers, such as HO‐1, SOD2, and ATPB, as well as mitochondrial genes, including mtDNA copy number and PGC‐1α. Additionally, the increased levels of inflammatory factors, including NF‐κB, TNF‐α, and IL‐1β, were remarkably attenuated by UMB treatment. These results suggest that UMB exerts renoprotective effects by activating NRF2 signaling to inhibit oxidative stress and inflammation, to some extent, in cisplatin‐induced AKI. However, the mechanism by which NRF2 signaling regulates inflammation and oxidative stress in cisplatin‐induced AKI remains unclear and requires further investigation.

In conclusion, our results further confirmed the protective effect of UMB against cisplatin‐induced AKI and demonstrated that these effects can be attributed to the activation of NRF2 signaling‐mediated inhibition of oxidative stress and inflammation. More importantly, as a natural product with high efficacy and low toxicity, UMB has potential implications in drug combinations and natural supplements for cisplatin‐induced AKI, thus providing a theoretical basis for the development of novel, comprehensive treatment approaches for AKI.

## CONFLICT OF INTEREST STATEMENT

The authors declare that there is no duality of interest associated with this manuscript.

## Ethics Statement

The study was approved by the Institutional Animal Care and Use Committee of Nanjing Medical University (registration number: IACUC 14030112–2).

## Supporting information


**Data S1.** Supporting Information.

## Data Availability

We can provide the required Data Availability Statement information in the manuscript, if necessary.
