# Supplementary material for: Umbelliferone attenuates cisplatin‐induced acute kidney injury by inhibiting oxidative stress and inflammation via NRF2
Source: Physiol Rep. 2023 Nov 29;11(23):e15879. doi: 10.14814/phy2.15879 (PMC10686806; doi:10.14814/phy2.15879)
Supplement: Supplementary file 1 — Data S1. Supporting Information. [file PHY2-11-e15879-s001.zip › PHYSREP-2023-07-231-T-s01.pdf]

Umbelliferone attenuates cisplatin-induced acute kidney injury by inhibiting oxidative stress and inflammation via NRF2

METHODS

In vivo

administered UMB by oral gavage

cisplatin injection into C57BL/6J mice

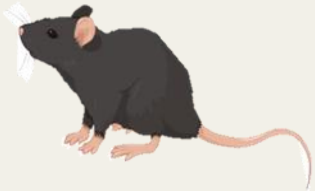

In vitro

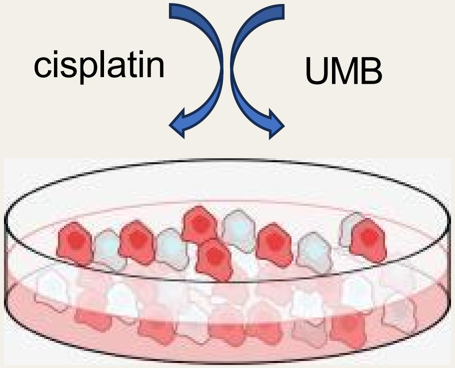

- Oxidative stress and inflammation
- Apoptosis
- Signaling pathways

OUTCOME

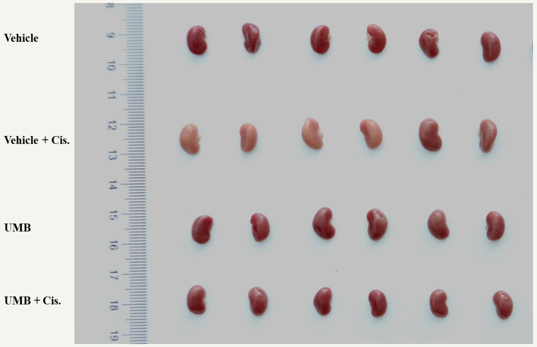

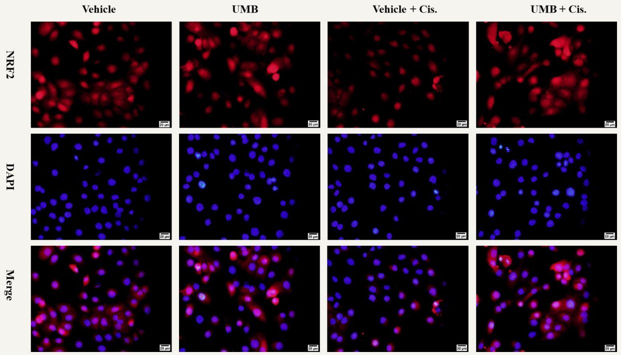

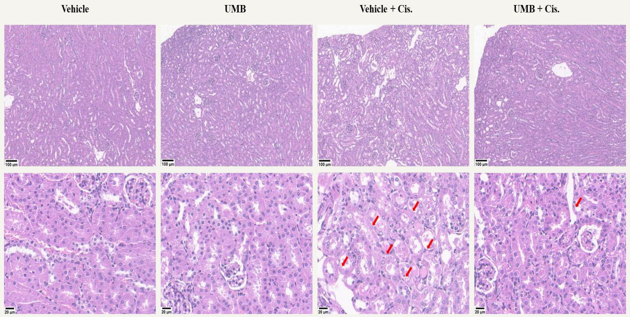

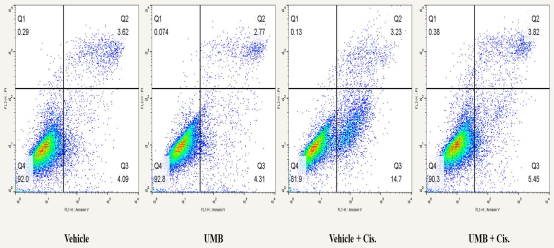

CONCLUSION Our results demonstrated that UMB can protect against cisplatin-induced nephrotoxicity, which was mediated by the NRF2 signaling pathway via antioxidant and anti-inflammatory activities. Therefore, UMB can be used in the clinical treatment of AKI.
