# Supplementary figures and images for: Umbelliferone attenuates cisplatin‐induced acute kidney injury by inhibiting oxidative stress and inflammation via NRF2
Source: Physiol Rep. 2023 Nov 29;11(23):e15879. doi: 10.14814/phy2.15879 (PMC10686806; doi:10.14814/phy2.15879)

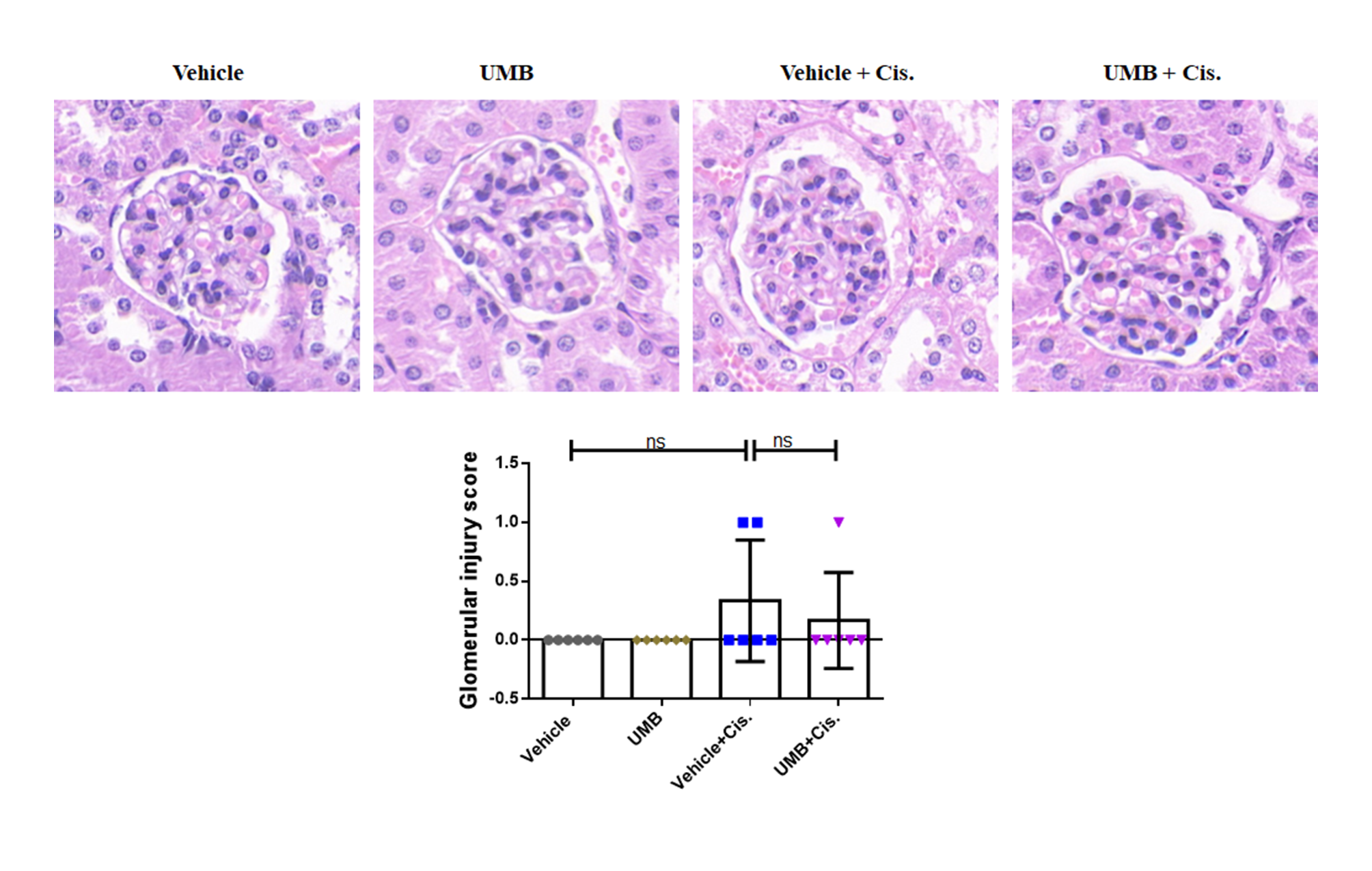

Supplement: Supplementary file 1 — Data S1. Supporting Information. [file PHY2-11-e15879-s001.zip › PHYSREP-2023-07-231-T-s02.tif]

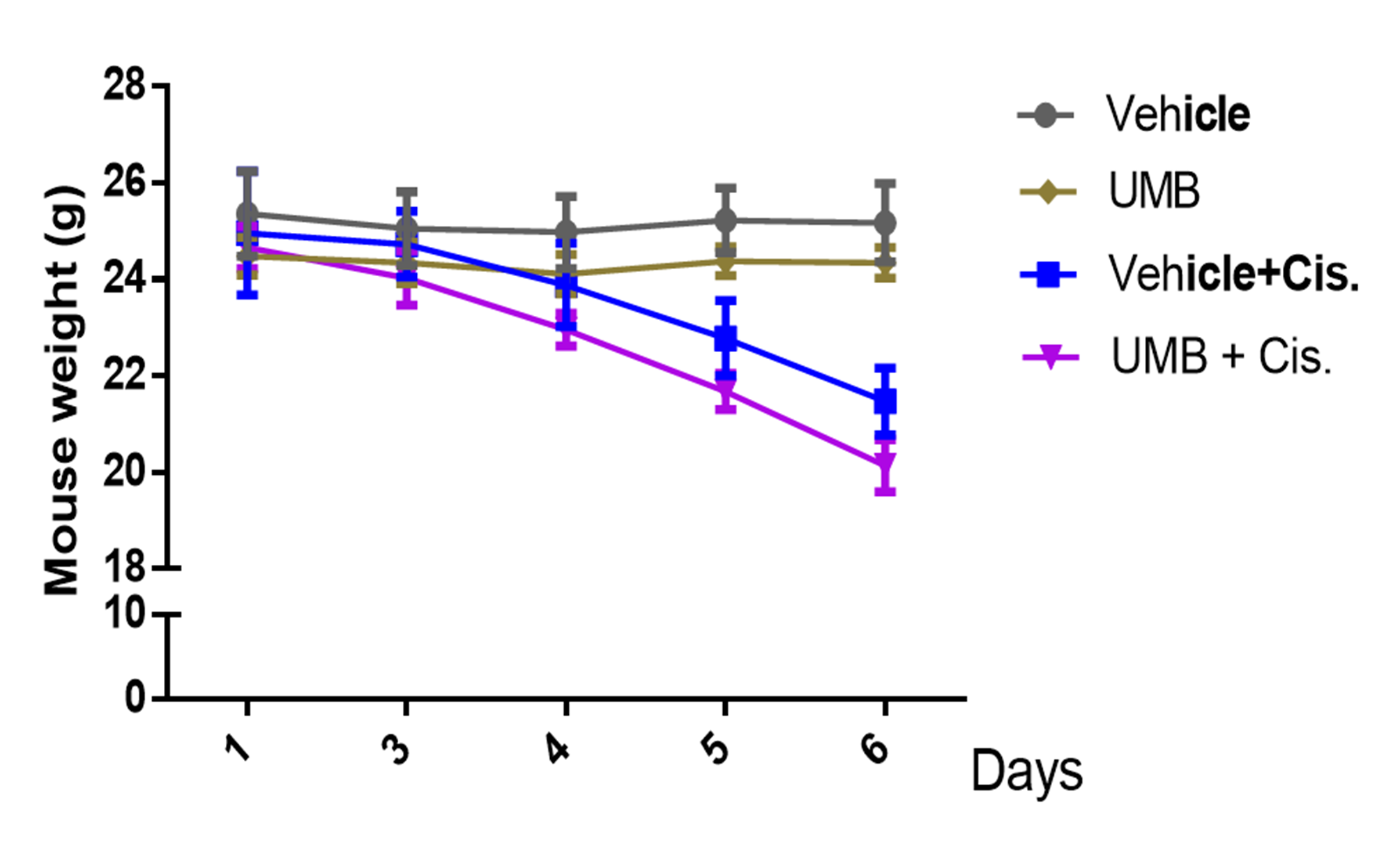

Supplement: Supplementary file 1 — Data S1. Supporting Information. [file PHY2-11-e15879-s001.zip › PHYSREP-2023-07-231-T-s03.tif]
